# Impact of Comorbidity and Prescription Drugs on Haemorrhage in Cholecystectomy

**DOI:** 10.1007/s00268-017-3961-3

**Published:** 2017-03-06

**Authors:** J. Strömberg, G. Sandblom

**Affiliations:** Department of Clinical Science, Intervention and Technology (CLINTEC), Karolinska Institutet, Stockholm, Sweden

## Abstract

**Background:**

The purpose of the present study was to analyse the impact of patient-related risk factors and medication drugs on haemorrhagic complications following cholecystectomy.

**Methods:**

All cholecystectomies registered in the Swedish population-based Register for Gallstone Surgery and ERCP (GallRiks) were identified. Risk factors for bleeding were assessed by linking data in the GallRiks to the National Patient Register and the Prescribed Drug Register, respectively. The risk of haemorrhage leading to intervention was determined by variable regression, and Kaplan–Meier analysis assessed survival rate following perioperative haemorrhage.

**Results:**

A total of 94,557 patients were included between 2005 and 2015, of which 799 (0.8%) and 1192 (1.3%) patients were registered as having perioperative and post-operative haemorrhage, respectively. In multivariable analysis, an increased risk of haemorrhagic complications was seen in patients with cerebrovascular disease (*p* = 0.001), previous myocardial infarction (*p* = 0.001), kidney disease (*p* = 0.001), heart failure (*p* = 0.001), diabetes (*p* = 0.001), peripheral vascular disease (*p* = 0.004), and obesity (*p* = 0.005). Prescription of tricyclic antidepressant (*p* = 0.018) or dipyridamole (*p* = 0.047) was associated with a significantly increased risk of perioperative haemorrhage. However, this increase in risk did not remain significant following Bonferroni correction for mass significance. Perioperative haemorrhage increased the risk of death occurring within the first post-operative year [Hazard Ratio, (HR) 4.9, CI 3.52–6.93] as well as bile duct injury (OR 2.45, CI 1.79–3.37).

**Conclusion:**

The increased risk of haemorrhage associated with comorbidity must be taken into account when assessing patients prior to cholecystectomy. Perioperative bleeding increases post-operative mortality and is associated with an increased risk of bile duct injury.

## Introduction

Laparoscopic cholecystectomy (LC) has since its introduction in the 1990s become the golden standard for the surgical treatment of symptomatic gallstone disease. In Sweden, about 13,000 cholecystectomies are performed each year of which 90% are performed with laparoscopic technique [1]. In LC, bile duct injuries and haemorrhage are the most feared complications. However, major haemorrhage is a rare event (0.08–0.25%) and is most often caused by injury to the right hepatic artery or inadvertent placement of a trocar in the aorta or vena cava [2–4]. More frequently occurring are minor bleeds arising from the abdominal wall and the liver bed following cholecystectomy (2.0–4.1%) [5, 6]. Haemorrhage from small vessels complicates the surgical procedure, increases the risk of conversion [7], and in some cases requires reoperation and/or blood transfusion [5].

Risk scoring systems based on comorbidity has been developed in order to predict bleeding risk. Furthermore, conditions such as renal and heart failure have been found to be associated with increased risk of haemorrhagic complications [8, 9]. In order to minimize the risk of procedure-related bleeding, drugs with anticoagulant or antiaggregant effects are usually discontinued. However, not often considered there are other drugs with side effects that might inadvertently affect platelet function and/or coagulation factors leading to impaired clotting ability [10, 11].

The influence of patient comorbidity and drugs on haemorrhagic complications in cholecystectomy is insufficiently known. We therefore undertook a population-based study to assess comorbidity as an independent risk factor for haemorrhagic complications. Furthermore, in the context of reducing haemorrhagic complications, a second part of the study aimed to investigate the need for the discontinuation of prescription drugs prior to cholecystectomy.

## Methods

### Study design

All laparoscopic and open cholecystectomy procedures registered between 2006 and 2015 in the Swedish Register for Gallstone Surgery and ERCP (GallRiks) [12] were included in the study. Every Swedish citizen has a unique National Registration Number (NRN) [13]. In order to assess the impact of prescribed drugs on risk of haemorrhage, the NRN was used as an aid in cross-matching the GallRiks with the Swedish Prescribed Drug Register (PDR; Swedish: Läkemedelsregistret) [14]. Only drugs prescribed to patients within 90 days prior to surgery were included in the analysis. The association of comorbidity with risk of bleeding was studied by comparing data from the GallRiks with the National Patient Register (NPR; Swedish: Patientregistret). Data in GallRiks were linked to the Swedish Death Register to obtain ICD codes indicating cause of death. Mortality was only considered to be procedure-related if a complication was registered on the death certificate as primary or contributing diagnosis.

### Definitions

Data regarding perioperative haemorrhage were obtained from the GallRiks. Perioperative haemorrhage was defined as blood loss during the procedure that led to conversion to open surgery, blood transfusion, or other intervention not routinely applied in gallstone surgery. A post-operative haemorrhage was defined as bleeding within 30 days after cholecystectomy that necessitated reoperation, blood transfusion, or resulted in prolonged hospital stay (>1 day longer than the median stay). The post-operative incidence was based on those patients registered in the GallRiks and/or PAR as having had a post-operative haemorrhage. The occurrence of haemorrhage in the peri- and/or post-operative period within 30 days following cholecystectomy was given the term “total bleed”. Bile duct injury was defined as injury and/or clinically significant leakage detected in the peri- and/or post-operative period.

### Data sources

The main purpose of the GallRiks is to assemble information, serve as a base for quality assurance, and facilitate research regarding gallstone surgery and ERCP. The register was started in 2005 and has today reached full national coverage (>90%). There are currently 79 hospitals/units in Sweden enrolled in the GallRiks. Repeated annual validation assures high-quality data with correctness in 97.2–98.2% of all registrations [15]. Data assembly in the GallRiks have previously been described in detail [16].

The population-based PDR has registered dispensed medication in Sweden since July 2005. All drugs prescribed are classified according to the Anatomical Therapeutic Chemical (ATC) classification system. Information on dose and date of dispensing of all prescribed drugs dispensed at Swedish pharmacies is registered in the PDR. However, the register contains no information regarding drugs administered during in-hospital treatment, over-the-counter (OTC) drugs (without prescription), or drugs administered in day care. In July–December 2005, the PDR registered 84% of the nation’s total consumption of drugs and 77% of the total expenditure [17].

The NPR was founded in 1964 and provides information and discharge diagnosis for all hospital admissions and outpatients in Sweden. With nationwide coverage, the NPR contains information on over 50 million patients since its inception. Diagnoses in the NPR are coded according the ICD system and currently over 99% of all discharges are covered [18]. The validity of the register has been tested by Ludwigsson et al. [19] showing a high accuracy (85–95%) for most diagnoses.

### Statistical analysis

The Chi-square test was used to compare patient characteristics. Univariate analysis was undertaken in order to identify predictors of haemorrhage with estimates of odds ratio (OR) and 95 per cent confidence intervals. In order to restrict confounding to a minimum, all variables in the multivariate regression analyses of patient characteristics were adjusted for gender, age, ASA, surgical indication, operative approach, thromboembolism prophylaxis, operation time, and prescribed anticoagulant medication.

The impact of prescribed drugs on bleeding was assessed by univariate regression.

In this analysis, mass significance was managed by Bonferroni correction [20], where data were considered significant at the 95% level if the *p* value divided by the number of drugs in the analysis (*n* = 28) was less than *p* = 0.05/28 = 0.002. Kaplan–Meier analysis assessed the overall survival following a registered haemorrhagic complication. Hazard ratios (HRs) were estimated using the Cox regression model. Gender, age, indication, operative approach, and ASA classification were added as covariables in the calculation of adjusted HR. All statistical analyses were performed using SPSS, IBM^®^ (version 23.0.0.0).

## Results

### Incidence

Altogether 94,557 patients registered in the GallRIks undergoing cholecystectomy (laparoscopic as well as open) 2005–2015 were included in the study. The total incidence of post-operative complications was 8.4% (7975 patients). Perioperative haemorrhage was registered in 799 (0.8%). The incidence of post-operative haemorrhage was 1.3% (1192 patients). There was 46.8% agreement in frequency of post-operative haemorrhage between the registers. The majority of post-operative complications registered in the GallRiks occurred within 30 days after surgery (*n* = 779, 97%). Bleeding was also registered between 30 and 60 days (*n* = 18, 2.25%) and between 60 and 90 days (*n* = 6, 0.75%). In total, 1867 patients were registered with a peri- and/or post-operative intervention due to haemorrhage, resulting in a total bleeding incidence of 2.0%. Haemorrhagic shock was registered in 10 patients (0.01%).

### Risk factors

Risk factors for haemorrhage in univariate regression (Table 1) were: male gender; high age; ASA > I; gallstone complications; conversion to open surgery; open cholecystectomy; thromboembolism prophylaxis; and long operation time. A subgroup analysis was conducted on 36,797 patients for whom BMI was registered (missing = 57,760). There were 12,334 patients (33.5%) with BMI < 18.5 (underweight), 13,776 patients (37.4%) with BMI 18.5–24.9 (normal weight), 9702 (26.4%) patients with BMI 25–29.9 (overweight), and 985 patients (2.7%) with BMI > 30 (obesity). The odds ratio for BMI ≥ 25 and bleeding was 1.04, (CI 0.89–1.23, data not shown). However, in the multivariate analysis, obesity was a significant risk factor for bleeding (OR = 1.3, CI 1.10–1.56).

**Table 1:** Patient characteristics as described by Chi-square test and risk of haemorrhage assessed by univariate regression

	*M*	*N* (%)	Periop bleed (%)	OR CI		Postop bleed (%)	OR CI		Total bleed^1^ (%)	OR CI	
Gender	1										
Female		63,488 (67.1)	408 (0.62)	1	Ref	697 (1.10)	1	Ref	1036 (1.63)	1	Ref
Male		31,068 (32.9)	391 (1.31)	2.15	1.87–2.47	495 (1.59)	1.46	1.30–1.64	831 (2.67)	1.66	1.51–1.82
Age	1										
≤40		28,457 (30.1)	117 (0.41)	1	Ref	273 (0.96)	1	Ref	378 (1.33)	1	Ref
41–60		37,252 (39.4)	243 (0.65)	1.59	1.28–1.98	383 (1.03)	1.07	0.92–1.25	590 (1.58)	1.20	1.05–1.36
>60		28,847 (30.5)	439 (1.52)	3.74	3.05–4.59	536 (1.86)	1.95	1.69–2.26	899 (3.12)	2.39	2.12–2.70
ASA	0										
I		49,172 (52.0)	258 (0.52)	1	Ref	434 (0.88)	1	Ref	660 (1.34)	1	Ref
II		38,094 (40.3)	390 (1.02)	1.59	1.28–1.98	534 (1.40)	1.07	0.92–1.25	871 (2.29)	1.20	1.05–1.36
≥III		7291 (7.71)	151 (2.07)	3.74	3.05–4.59	224 (3.07)	1.95	1.69–2.26	336 (4.61)	2.39	2.12–2.70
Indication	0										
Gallstone colic		54,681 (57.8)	312 (0.57)	1	Ref	518 (0.95)	1	Ref	791 (1.45)	1	Ref
Gallstone complications^2^		36,659 (38.8)	454 (1.24)	2.19	1.89–2.53	606 (1.65)	1.76	1.56–1.98	986 (2.69)	1.88	1.71–2.07
Acalculous cholecystitis		648 (0.70)	4 (0.62)	1.08	0.40–2.91	25 (3.90)	4.20	2.79–6.32	28 (4.32)	3.08	2.09–4.52
Other		2569 (2.70)	29 (1.13)	1.99	1.36–2.92	43 (1.67)	1.78	1.30–2.44	62 (2.41)	1.69	1.30–2.19
Op approach	2293										
Lap		78,814 (85.4)	356 (0.45)	1	Ref	746 (0.95)	1	Ref	1066 (1.35)	1	Ref
Converted to open surgery		6593 (7.20)	272 (4.13)	9.48	8.08–11.13	214 (3.25)	3.51	3.01–4.10	433 (6.57)	5.13	4.57–5.75
Open		6857 (7.40)	155 (2.26)	5.10	4.21–6.17	187 (2.73)	2.93	2.50–3.45	307 (4.48)	3.42	3.00–3.89
Thromboembolism prophylaxis	289										
No		57,696 (61.2)	273 (0.47)	1	Ref	534 (0.93)	1	Ref	758 (1.31)	1	Ref
Yes		36,572 (38.8)	526 (1.44)	3.07	2.65–3.56	643 (1.76)	1.92	1.71–2.15	1094 (3.0)	2.32	2.11–2.54
Op time	290										
<120		71,552 (75.9)	288 (0.40)	1	Ref	750 (1.05)	1	Ref	1001 (1.40)	1	Ref
>120		22,715 (24.1)	511 (2.25)	5.62	4.86–6.50	428 (1.88)	1.85	1.64–2.08	852 (3.75)	2.76	2.51–3.02

^1^Total bleed perioperative and/or post-operative haemorrhage and/or haemorrhagic shock)

^2^Cholecystitis and/or gallstone pancreatitis

*M* missing data, *N* number of patients, *OR* odds ratio, *CI* 95% confidence interval, *Ref* reference

In Table 2, 28 prescription drugs with known anticoagulant or antiplatelet effect were tested for association with bleeding. In the univariate analysis, dipyridamole (*n* = 252, OR = 2.39, CI 0.98–5.81) and tricyclic antidepressants (TCA, *n* = 1151. OR = 1.78, CI 1.10–2.89) had a significantly elevated risk of perioperative haemorrhage. This increase in risk, however, did not remain significant in a Bonferroni correction.

**Table 2:** Prescribed drugs^1^ and haemorrhagic complications

Drug	*N* (%)	Periop bleed (%)	*p*	Postop bleed	*p*	Total bleed^2^ (%)	*p*
Acetysalicylic acid	7443 (8.75)	61 (8.5)	0.81	106 (9.68)	0.27	159 (9.36)	0.36
ACE inhibitor	6575 (7.73)	53 (7.38)	0.73	80 (7.31)	0.60	126 (7.42)	0.63
Apixaban	11 (0.01)	0 (0)	n.s	0 (0)	n.s	0 (0)	n.s
Azathioprin	179 (0.21)	0 (0)	n.s	0 (0)	n.s	0 (0)	n.s
Acyclovir	198 (0.23)	2 (0.28)	0.80	2 (0.18)	0.22	4 (0.24)	0.98
Beta-lactam antibiotics	4790 (5.63)	51 (7.10)	0.09	63 (5.75)	0.86	101 (5.95)	0.56
Calcium channel inhibitor	6146 (7.22)	60 (8.40)	0.24	76 (6.94)	0.72	130 (7.66)	0.49
Carboxamide	361 (0.42)	1 (0.14)	0.24	2 (0.18)	0.22	3 (0.18)	0.11
Cephalosporin	31 (0.04)	0 (0)	n.s	1 (0.10)	0.34	1 (0.06)	0.62
Clopidogrel	565 (0.66)	7 (0.97)	0.30	4 (0.37)	0.22	11 (0.65)	0.93
Cytostatic agents	238 (0.28)	3 (0.42)	0.48	6 (0.55)	0.09	7 (0.41)	0.30
Dabigatran	37 (0.04)	1 (0.14)	0.22	0 (0)	n.s	1 (0.06)	0.76
Dipyridamole	252 (0.30)	5 (0.70)	0.05*	1 (0.10)	0.21	6 (0.35)	0.66
Heparin	838 (0.98)	7 (0.97)	0.98	11 (1.0)	0.95	15 (0.88)	0.67
Kinin	58 (0.07)	0 (0)	n.s	0 (0)	n.s	0 (0)	n.s
NSAID	7932 (9.32)	73 (10.2)	0.43	102 (9.32)	1.0	163 (9.60)	0.69
Phenytoin	77 (0.09)	0 (0)	n.s	2 (0.18)	0.31	2 (0.12)	0.71
Prasugrel	5 (0.01)	0 (0)	n.s	0 (0)	n.s	0 (0)	n.s
Rifampicin	16 (0.02)	0 (0)	n.s	1 (0.10)	0.08	1 (0.06)	0.22
Rivaroxaban	23 (0.03)	0 (0)	n.s	0 (0)	n.s	0 (0)	n.s
SSRI	5203 (6.11)	45 (6.27)	0.86	67 (6.12)	0.99	106 (6.24)	0.82
Statin	7377 (8.67)	58 (8.10)	0.57	108 (9.86)	0.16	157 (9.25)	0.39
Thiazide	2355 (2.77)	25 (3.48)	0.24	31 (2.83)	0.90	55 (3.24)	0.23
Ticagrelor	32 (0.04)	0 (0)	n.s	1 (0.10)	0.36	1 (0.06)	0.65
Tricyclic antidepressant	1151 (1.35)	17 (2.37)	0.02*	18 (1.64)	0.40	32 (1.88)	0.055
Trimethoprim/sulphamethoxazole	255 (0.30)	4 (0.56)	0.21	4 (0.37)	0.69	6 (0.35)	0.68
Valproate	210 (0.25)	2 (0.28)	0.86	0 (0)	n.s	2 (0.12)	0.28
Warfarin	1818 (2.14)	21 (2.92)	0.14	27 (2.47)	0.45	45 (2.70)	0.14

Table 2: Pearson´s Chi-Square test

* Significant within 95% confidence interval

^1^Drugs prescribed within 90 days prior to surgery

^2^Total haemorrhage (peri- and/or post-operative) for each drug

Cerebrovascular disease (OR = 1.96; CI 1.64–2.34), previous myocardial infarction (OR = 1.94, CI 1.56–2.40), kidney disease (OR = 1.93; CI 1.41–2.63), heart failure (OR = 1.73, CI 1.37–2.18), diabetes (OR = 1.49, CI 1.27–1.74), peripheral vascular disease (OR = 1.49, CI 1.13–1.95), and obesity (OR = 1.27, CI 1.08–1.51) were all found to be associated with a significantly increased risk of haemorrhagic complications (Table 3).

**Table 3:** Comorbidity and haemorrhagic complications^1^

Condition	*N* (%)	Bleed (%)	Univariate			Multivariate		
			*p*	OR	CI	*p*	OR	CI
Cerebrovascular disease	3283 (3.48)	151 (4.60)	0.001	2.52	2.12–2.98	0.001	1.96	1.64–2.34
Diabetes	5396 (5.71)	197 (3.65)	0.001	1.99	1.71–2.31	0.001	1.49	1.27–1.74
Heart failure	1797 (1.90)	103 (5.73)	0.001	3.14	2.56–3.85	0.001	1.73	1.37–2.18
Kidney disease	788 (0.83)	48 (6.10)	0.001	3.28	2.44–4.41	0.001	1.93	1.41–2.63
Liver cirrhosis	345 (0.37)	14 (4.10)	0.005	2.11	1.23–3.61	0.053	1.71	0.99–2.94
Lung disease	8750 (9.27)	181 (2.10)	0.51	1.05	0.90–1.23	0.363	0.930	0.79–1.09
Obesity	6173 (6.54)	157 (2.54)	0.01	1.32	1.12–1.56	0.005	1.27	1.08–1.51
Peripheral vascular disease	1314 (1.40)	64 (4.87)	0.001	2.60	2.01–3.35	0.004	1.49	1.13–1.95

^1^Peri- and/or post-operative haemorrhagic complications

### Bile duct injury

By including perioperative iatrogenic bile duct injury and/or post-operative bile leakage, the overall incidence of bile duct injury was 1.6%. For patients with perioperative haemorrhage, the incidence of bile duct injury was significantly higher (5.4%, *p* < 0.05). Adjusting for gender, age, indication, operative approach, and ASA classification, perioperative bleeding was found to be a risk factor for bile duct injury (OR = 2.45, CI 1.79–3.37).

### Mortality

**Fig. 1:**
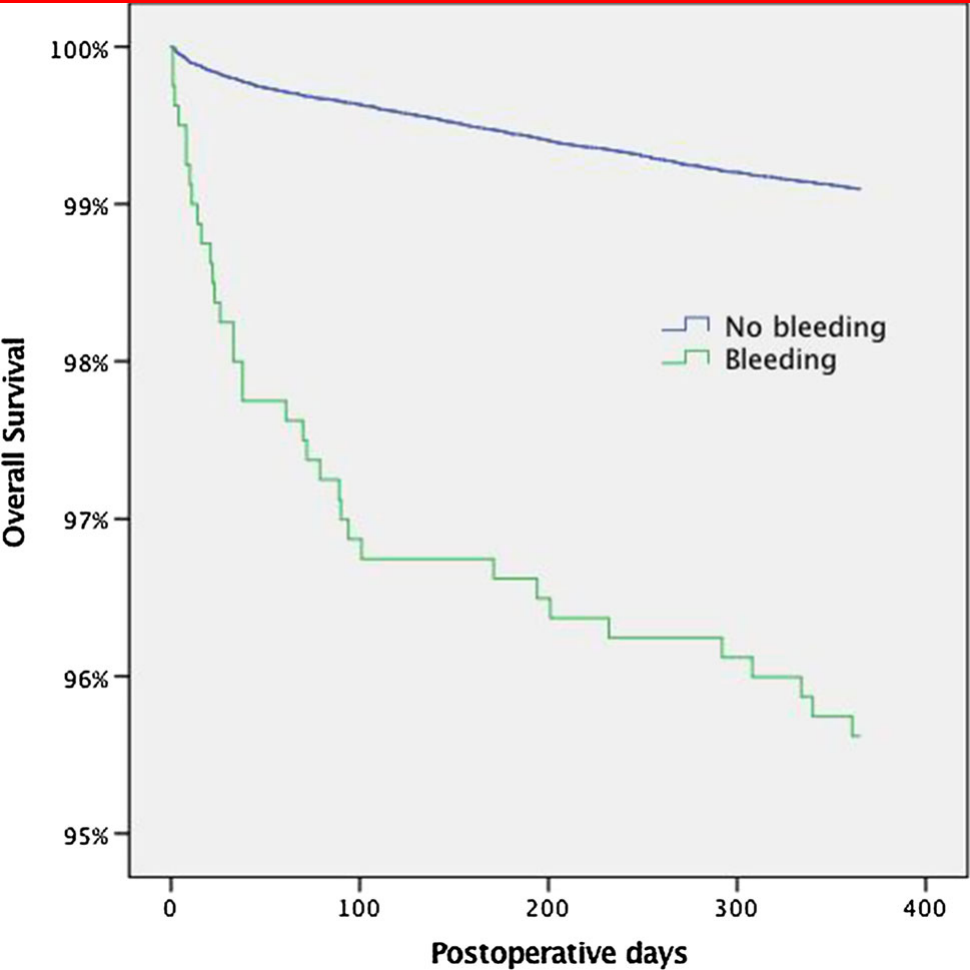
Kaplan-Meier plot of survival following peri-operative haemorrhage in cholecystectomy

In total, 223 (0.24%) patients died within 30 days after cholecystectomy. The cause of death was procedure-related in 25 (0.03%). In patients with perioperative, post-operative, and total bleeding, 16 patients (2.0%), 25 patients (11.2%), and 34 patients (15.2%) died, respectively. Almost half of patients (48%) with a procedure-related cause of death had a haemorrhage registered prior to death. Patients that had a perioperative haemorrhage had a significantly increased mortality during the first post-operative year (Fig. 1). The increased hazard of death (HR) associated with perioperative bleeding was estimated to be 4.9 (CI 3.52–6.93) with an adjusted HR of 1.8 (CI 1.28–2.53). The increased mortality in patients with a perioperative haemorrhage remained when performing a subgroup analysis where death within thirty days was excluded (*p* < 0.05).

## Discussion

In this population-based study, cerebrovascular disease, previous myocardial infarction, kidney disease, heart failure, diabetes, peripheral vascular disease, and obesity were all found to be independent risk factors for haemorrhagic complications in cholecystectomy. Furthermore, patients with perioperative haemorrhage had a fivefold increase in hazard ratio for post-operative death. Perioperative bleeding significantly increased the risk of bile duct injury.

### Strength and weakness

The strength that this study presents is the population-based design, with data retrieved from three validated Swedish quality registers. Other strengths are that haemorrhage was presented as perioperative, post-operative as well as total. Our definition of haemorrhage in this study was a bleed that mandated intervention. It could be argued, however, that a more stringent way of defining a haemorrhage would be a minimum volume of blood loss.

There are some potential weaknesses with the present study. Data on prescribed drugs did not provide information on if, or for how long the drug was discontinued prior to surgery. In this study, the assessment of potent anticoagulants such as Warfarin and new oral anticoagulants (NOACs) was difficult since it was unlikely to believe that these drugs would have been continued during surgery. Furthermore, the timing of surgery after interrupting the anticoagulant treatment may also affect the risk of bleeding. Since we do not have any data on this, we are not able to determine the optimal interval to perform surgery after anticoagulant cessation.

Another weakness was the lack of precision in comorbidity diagnoses. For instance, “kidney disease” is a group diagnosis made up from a number of kidney disorders. Furthermore, registers on surgical procedures that are based on self-reported adverse events are inevitably subject to selection bias since it is more likely that a positive outcome of surgery is reported. This effect is lessened by repeated validation. Surgical units enrolled in GallRiks are continuously visited by appointed inspectors, and during these validations, data from medical journals are scrutinized and compared with those entered in the register. Previous cross-check control of data has found less than 2% error [15].

### Comparison with other studies

The overall incidence of haemorrhage during and within 30 days after cholecystectomy was 2.0%. Due to differences in criteria, the comparison of haemorrhage rates between different studies must be interpreted with caution. Shea et al. [21] reported in a meta-analysis a bleeding complication rate of 10.5% and Z´graggen reported a perioperative haemorrhage incidence of 1.97% [7]. In a study by Ercan et al., patients taking oral anticoagulants (OACs) were taken off their medication prior to cholecystectomy and given enoxaparin (low molecular heparin) bridging therapy and compared to a control group. In the OAC group, the post-operative bleeding rate was 25% compared to 1.5% in the control group [22].

In accordance with previous studies [7, 21, 23, 24], we found that the overall 30-day mortality following cholecystectomy is low (0.24%). However, haemorrhage leading to intervention increased mortality by over 60 times compared to patients without bleeding complications. Furthermore, half of all patients that died in the post-operative period from procedure-related causes were registered as having a haemorrhage prior to death. A Kaplan–Meier estimate also showed that survival of patients who had a perioperative haemorrhage continued to decline beyond the 30-day post-operative interval.

Serotonin plays an important role in the process of platelet aggregation and vasoconstriction during primary haemostasis [25]. Indeed, by reducing the re-uptake of serotonin, TCA inhibits platelet aggregation [26] and has been associated with an elevated bleeding risk [27, 28]. In our study, tricyclic antidepressants (TCA) significantly increased the risk of perioperative haemorrhage. However, the correlation did not tolerate a Bonferroni correction, indicating that the result could be an artefact of mass significance. Nevertheless, TCA may have a clinically relevant impact on the risk of haemorrhage that should be evaluated in future studies.

Following cholecystectomy, it has been shown that a significant number of patients with bile duct injuries have an associated vascular injury [29, 30]. These “vasculobiliary” injuries are often managed at tertiary care facilities [30]. Risk factors for biliary injury during cholecystectomy have previously been described in detail [31]. However, to the best of our knowledge, perioperative bleeding has not been defined as a significant risk factor for bile duct injury. As previously reported, bile duct injuries may occur as a consequence of a bleeding-impaired exposure and visualization during dissection in cholecystectomy [32].

It is reasonable to believe that a perioperative haemorrhage distorts anatomical landmarks, resulting in an increased risk of iatrogenic bile duct injury or misplacement of clips. In order to account for all possible injuries to the bile ducts, we chose a broader definition for bile duct injury including both iatrogenic injuries detected during the operation and/or bile leaks in the post-operative phase. This resulted in a higher incidence of bile duct injuries than previously reported [4, 7]. In the present study, perioperative bleeding was associated with an increased incidence of injury and/or bile leakage. Moreover, in a multivariable regression model, perioperative bleeding was a strong predictor of bile duct injury.

## Conclusion

Patient comorbidity needs to be taken into account when assessing the risk of haemorrhage and peri-operative bleeding increases the risk of death following cholecystectomy. However, our results suggest that commonly prescribed medication does not need to be discontinued in order to reduce haemorrhagic complications. Finally, surgeons need to be aware of the increased risk of biliary duct injury following haemorrhage and should consider converting to open surgery if visualization and exposure is inadequate.
